# Opicapone Efficacy and Tolerability in Parkinson's Disease Patients Reporting Insufficient Benefit/Failure of Entacapone

**DOI:** 10.1002/mdc3.13094

**Published:** 2020-10-21

**Authors:** Petr Vokurka, Andrew Barron, Sheetal Sumaria, Lindsey Stockford, Paul Jarman, Kailash Bhatia, Simon Farmer, Tabish Saifee, Tom Warner, Rimona Weil, Sonia Gandhi, Patricia Limousin, Prasad Korlipara, Tom Foltynie

**Affiliations:** ^1^ Department of Clinical & Movement Neurosciences University College London Institute of Neurology London UK; ^2^ The National Hospital for Neurology and Neurosurgery, Queen Square London UK

**Keywords:** opicapone, entacapone, audit

## Abstract

**Background:**

Opicapone, a recently introduced catechol‐o‐methyl transferase (COMT) inhibitor has the advantage of being administered once daily, and has pharmacokinetic data to indicate it offers a greater degree of COMT inhibition than entacapone. Although trial data indicate it is non‐inferior to entacapone, there are no data to indicate whether it offers any clinical advantages.

**Methods:**

In this audit, we present data from 57 individuals prescribed opicapone at the National Hospital for Neurology and Neurosurgery, Queen Square who had either not tolerated or reported insufficient benefit following previous prescription of entacapone.

**Results:**

A total of 20 of 57 patients switched directly from entacapone to opicapone (“entacapone switchers”) whereas 37 of 57 patients had previously discontinued entacapone because of lack of benefit or adverse events (“entacapone failures”). A total of 21 of 57 (37%) patients stopped opicapone prior to 6 months. A total of 7 of 20 (35%) “entacapone switchers” experienced adverse events with opicapone of which 5 stopped the drug prior to the 6 month evaluation of efficacy. A total of 23 of 37 (62%) “entacapone failures” reported adverse events of which 16 stopped the drug. Among 36 of 57 (63%) patients who continued to use opicapone at 6 months, there was an improvement in OFF time of ~2 hours per day as measured by interview.

**Conclusions:**

We conclude that opicapone can be an effective additional treatment for wearing off in Parkinson's disease (PD) in a subgroup of patients. The use of opicapone in our cohort with prior entacapone exposure, however, was associated with higher rates of adverse effects and treatment discontinuation than reported in published trial data of COMT inhibitor naïve patients.

Motor fluctuations including “wearing off” are a major problem in the management of Parkinson's disease (PD) patients.^1^ Options include using additional doses of levodopa (l‐dopa), adding accessory drugs to the l‐dopa regime such as catechol‐o‐methyl transferase (COMT) inhibitors or monoamine oxidase‐B (MAO‐B) inhibitors, using short‐ or long‐ acting dopamine agonist medications or advanced therapies such as deep brain stimulation (DBS), apomorphine or l‐dopa/carbidopa intestinal gel.^2^, ^3^, ^4^

Until recently, the only COMT inhibitors licensed for PD were entacapone and tolcapone. Despite tolcapone showing an improvement in ON time (15%) compared with entacapone (8%), the use of tolcapone has diminished greatly because of concerns about hepatotoxicity and the requirement for regular liver function monitoring.^5^ Entacapone is used routinely in the management of PD but has a short half‐life requiring multiple daily dosing. Entacapone also frequently causes diarrhea and has the unfortunate side effect of turning bodily secretions an orange color.^6^ A proportion of patients taking entacapone also continue to report problematic OFF periods for which new therapeutic options are required.

Opicapone, a once daily COMT inhibitor, was licensed in Europe in 2016 and the United States in 2020 as an adjunctive drug to l‐dopa in patients with Parkinson's disease and end‐of‐dose motor fluctuations.^7^, ^8^, ^9^ It demonstrated superiority to placebo and non‐inferiority to entacapone in COMT inhibitor naïve patients (BIPARK 1).^10^ Although this study was not designed to test superiority of opicapone to entacapone, a favorable non‐significant reduction in OFF time was reported. This encouraging result follows pharmacokinetic data showing opicapone provides sustained and higher COMT inhibition than entacapone.^11^ In a follow‐up extension of the BIPARK 1 study, patients switching from entacapone to opicapone experienced an additional reduction in OFF time.^7^, ^12^

There are no studies designed to assess superiority of opicapone over entacapone, and only limited data on the safety and efficacy of opicapone among COMT inhibitor pre‐treated patients.^7^ Encouraged by the theoretical advantages of opicapone observed in pharmacokinetic studies and in COMT inhibitor naïve patients in the BIPARK studies, the National Hospital for Neurology and Neurosurgery (NHNN) planned an evaluation of the introduction of opicapone into clinical practice for patients experiencing disabling end‐of‐dose motor fluctuations who had not benefitted adequately from the use of entacapone. Here, we present the results of this evaluation.

## Methods

This was a single‐center, observational, prospectively planned audit of opicapone use in the specialist hospital management of PD patients experiencing disabling end‐of‐dose motor fluctuations despite prior treatment with entacapone. Outcomes of interest were efficacy (daily OFF time), safety, and tolerability.

Prior to prescribing opicapone, prescribers documented patient total daily OFF time, and estimated typical duration of ON time following each dose of l‐dopa, based on each patient's and their carer's joint best estimates. Patients were told that they might need to adjust the dose or timing of their l‐dopa regime if they developed dyskinesia but this was not systematized.

Repeat self‐reported OFF and ON times following direct questioning was again documented after 6 months. Audit data was subsequently collected from hospital medical notes in addition to basic demographic variables (sex and age), treatment start date, disease duration, indication, and treatment regimen pre‐opicapone and 6 months post‐opicapone. Any missing data from medical records was supplemented by phone calls to patients. Best clinical judgment was used to resolve any discrepancies between patient reported data and clinical records. All patients were asked open questions about any adverse events related to exposure to opicapone and whether these events led to its cessation.

All patients who were prescribed opicapone from January 2017 until December 2018 were included in this evaluation. This was an audit of the outcomes of opicapone prescription, based on the clinical need of the patients and conducted by the clinicians directly responsible for the clinical management of their own patients. As such formal ethical approval was not deemed necessary, however the principles of the Declaration of Helsinki and all aspects of Good Clinical Practice were followed at all times.

Quantitative data were analyzed using IBM SPSS statistical package, version 25 (IBM Corp., USA). Given that our focus of interest was (1) the tolerability of opicapone, and (2) the efficacy of opicapone among those who continued to be prescribed the drug, we chose to analyze the efficacy data per protocol, ie, in accordance with whether patients continued to use opicapone rather than present an intention to treat analysis. The difference in OFF time between pre‐ and post‐opicapone was tested by a paired *t* test. Mann‐Whitney *U* tests were performed for non‐parametric data comparisons. Baseline variables that differed in univariate analyses between those patients who continued opicapone verses those that stopped opicapone at a relaxed *P* value of 0.2 were included in a multivariate logistic regression analysis.

## Results

### Baseline Characteristics

**FIG 1:**
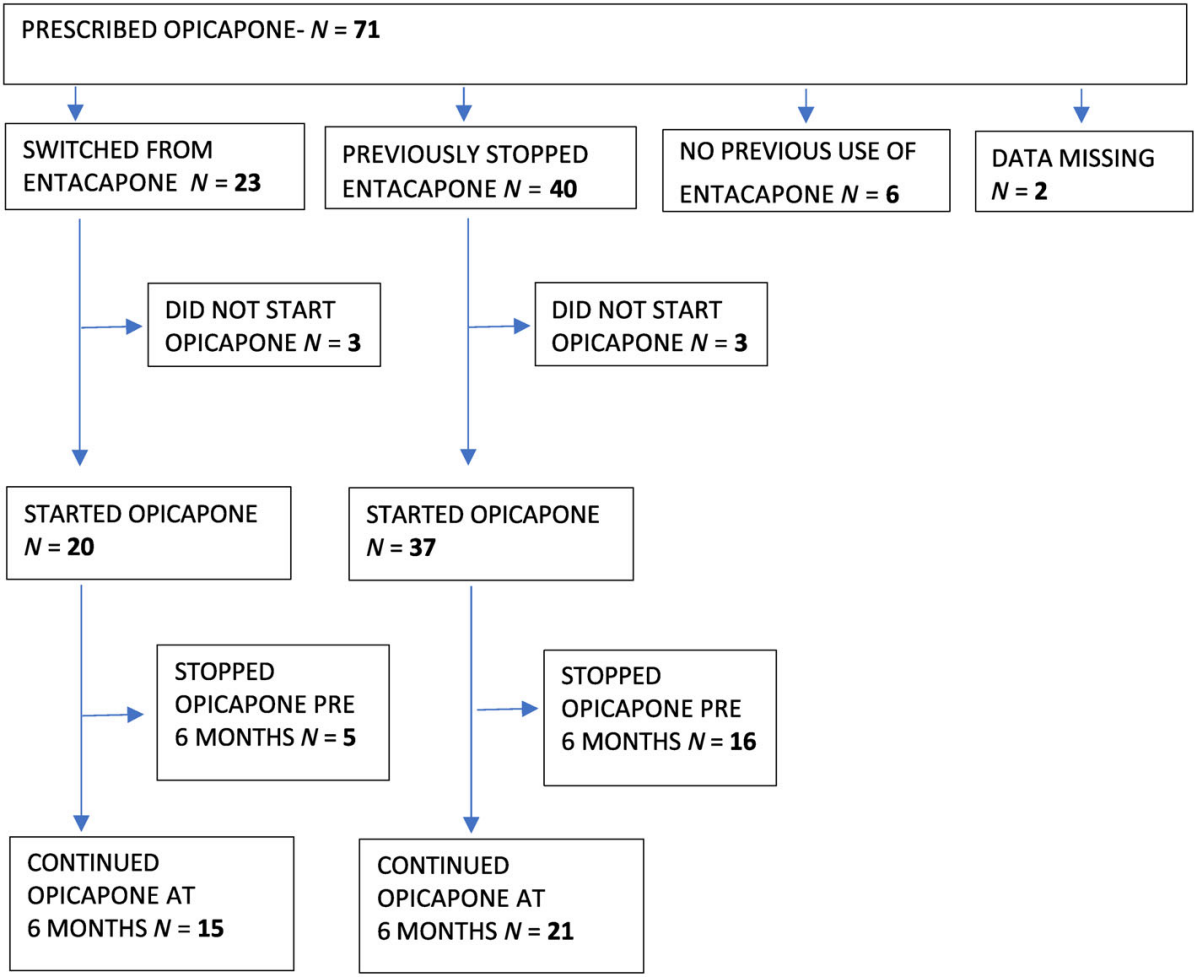
Flowchart of patients included in the study.

Opicapone was prescribed for 71 patients of whom 62 individuals commenced treatment. This included 20 patients using entacapone up until the time of opicapone introduction and 37 patients who had previously stopped entacapone because of lack of efficacy or lack of tolerability. Four patients who had never used entacapone, and 1 patient in whom data regarding previous entacapone use was unobtainable, were excluded from the analysis (Fig. 1).

The demographic data of the 57 patients who started opicapone according to the approved protocol are described in Table 1.

**TABLE 1:** *Baseline demographics of the 57 patients who started using opicapone per protocol and subgroups according to direct switch from etacapone or prior cessation of entacapone*

	All patients starting opicapone (n = 57; 32 male, 25 female) mean (range)	Patients who switched from entacapone (n = 20; 12 male, 8 female) mean (range)	Patients who had previously stopped entacapone (n = 37; 20 male, 17 female) mean (range)	Baseline comparison between entacapone switchers and prior stoppersmean difference (SE)*P* value
PD duration (yr)	11.0 (2–26)	10.0 (3–19)	11.6 (2–26)	1.5 (1.4) *P* = 0.28
Age (yr)	64.0 (45–84)	61.5 (45–73)	65.4 (47–84)	3.9 (2.6) *P* = 0.14
l‐dopa dose (mg/day)		950 (400–2150)	857 (225–1800)	*U* = 265^*^ *P* = 0.46
l‐dopa equivalent daily dose (mg/day)	1285 (475–2398)	1415 (782–2272)	1213 (475–2398)	*U* = 263^*^ *P* = 0.07
l‐dopa frequency (doses/day)	6.2 (3–9)	6.2 (4–9)	6.3 (3–9)	NS
OFF time (hr/day)	5. 3 (1.5–12)	5.0 (2–8)	5.4 (1.5–12)	0.4 (0.75) *P* = 0.61
ON time after each l‐dopa dose (hr/dose)	2.0 (0.25–4)	2.1 (1.25–3.5)	2.0 (0.25–4)	0.1(2.3) *P* = 0.61

^a^Non‐parametric comparisons were performed for comparisons of non‐normally distributed data.

### Duration of Use of Opicapone following Prescription

Of the 57 patients starting opicapone, 21 (37%) stopped the drug prior to 6 months with 12 stopping within the first month. A total of 36 of 57 patients who persisted with opicapone had a slightly shorter disease duration, were younger, and had less OFF time and longer periods of ON time than those who stopped opicapone (Table 2). Patients were categorized at baseline as either being switched directly from entacapone (“entacapone switchers”) or had previously stopped entacapone (“entacapone failures”). A total of 5 of 20 (25%) of entacapone switchers subgroup stopped opicapone prior to 6 months compared to 16 of 37 (43%) entacapone failures subgroup.

**TABLE 2:** *Baseline features of patients according to subsequent continuation/ cessation of opicapone*

	Continued opicapone beyond 6 months n = 36 (21 male) mean (SD)	Stopped opicapone prior to 6 months n = 21 (11 male) mean (SD)	Mean difference(SE)	*P* value
Direct switch from entacapone/previously stopped entacapone	15 (42%)/21(58%)	5 (24%)/16 (76%)	Pearson χ^2^ 1.9^§^	0.17
Age at opicapone use	62.7 (9.6)	66.2 (9.39)	3.6 (2.6)	0.18
Duration of PD	9.9 (4.4)	12.9 (5.7)	3.0 (1.4)	0.03
Baseline l‐dopa daily dosage (mg)	899 (408)	875 (418)	24*	0.72*
Baseline LEDD (mg)	1302 (490)	1255 (380)	47 (124)	0.71
Baseline OFF time (hr/day)	4.7 (2.0)	6.3 (3.3)	1.58 (0.72)	0.07
Baseline ON time (hr/l‐dopa dose)	2.3 (0.67)	1.7 (0.93)	0.53 (0.22)	0.02

^*^Mann‐Whitney *U* test.

^§^Pearson Chi squared test.

### Adverse Events Reported with Opicapone

The main reasons for patients discontinuing opicapone were related to adverse events. The main dopaminergic adverse events were the appearance of hallucinations and worsening of psychosis with behavioral changes, followed by dizziness and hyperhidrosis. Five patients reporting hallucinations developed these for the first time after opicapone was introduced, whereas in 2 patients hallucinations had already existed but became more pronounced with opicapone. One patient who experienced hallucinations also suffered from vivid dreams and another reported nightmares on opicapone. Although patients had been told that their l‐dopa dose might need adjusting after opicapone introduction, new onset hallucinations invariably led to immediate cessation of opicapone and on 2 occasions required hospital admission of the patient. Nausea (vomiting) and dyskinesia were each reported by 2 patients. Table 3 lists all adverse events documented in medical notes and/or reported by patients, separately according to whether they had directly switched from entacapone to opicapone or had previously stopped treatment with entacapone.

**TABLE 3:** *Adverse events reported following opicapone use, separately according to timing of entacapone cessation*

Type of adverse event	Patients who switched from entacapone reporting adverse event (n = 20)	Patients who had previously stopped entacapone reporting adverse event (n = 37)
Confusion	0	1
Depression	1	1
Dyskinesia	1	1
Disorientation	0	1
Dizziness	1	1
Tremor	0	1
Dystonia	0	1
Hallucinations	3	4
Mobility, gait decline	0	1
Nausea	0	2
Psychosis	0	2
Hyperhidrosis	0	1
Weight loss	1	0

Interestingly, 2 patients who suffered from poor fragmented sleep on opicapone improved their quality of sleep after opicapone was rescheduled in the morning. They did not stop opicapone. Neither diarrhea nor bodily fluid discoloration was reported by any patients.

### Efficacy of Opicapone among Ongoing Users

Data were available from 33 of 36 patients who continued on opicapone for at least 6 months. Among those who continued treatment, there was a significant decrease in daily OFF time from baseline (mean = 4.6 hr) to the 6‐month follow‐up (mean = 2.5 hr) resulting in a mean decrease in OFF time after 6 months of 2.1 hr, (95% confidence intervals [CI]: 1.3–2.80 hr; *P* < 0.00001).

Considering subgroups according to baseline entacapone use; the mean decrease in OFF time after 6 months was 2.8 hr (standard deviation [SD] = 2.6) in entacapone switchers, whereas entacapone failures experienced 1.6 hr (SD = 1.5) less daily OFF time, ie, a non‐significant mean difference in outcomes between groups of 1.1 hr of daily OFF time (*P* = 0.173).

There was a significant increase in estimated ON time following each dose of l‐dopa from baseline (mean = 2.3 hr), compared to 6‐month follow‐up (mean = 2.9 hr) resulting in a mean difference of 0.6 hr, (95% CI = 0.4–0.9 hr; *P* < 0.0001) following each dose of l‐dopa. Entacapone switchers experienced an improvement in ON time of 0.54 hr per dose of l‐dopa and entacapone failures experienced an improvement in ON time of 0.65 hr (*P* = 0.66).

### Factors Predictive of Opicapone Continuation/Cessation

Of the 57 patients, baseline factors that differed between those who continued opicapone beyond 6 months and those who discontinued opicapone were; disease duration at baseline, patients' age at baseline, daily OFF time in hours at baseline, ON time following l‐dopa dose, ongoing entacapone use at baseline (Table 2). These variables were all included in a logistic regression model to explore likelihood of continuation of opicapone at 6 months are described in Table 4. An increased amount of OFF time at baseline was significantly associated with an increased likelihood of discontinuing opicapone. The subgroup entacapone failures were more than 2.3 times more likely to discontinue opicapone than the entacapone switchers subgroup but this failed to reach statistical significance in multivariate analysis.

**TABLE 4:** *Factors predicting continuation/cessation of opicapone among 57 patients prescribed opicapone*

Variable	β coefficient	SE	*P* value	Exp (β)	CI Exp (β)	
					Lower	Upper
Age at opicapone onset (yr)	0.03	0.04	0.47	1.03	0.96	1.11
Disease duration at opicapone onset (yr)	0.12	0.07	0.08	1.13	0.98	1.30
Daily OFF time at baseline (hr)	0.22	0.13	0.09	1.24	0.97	1.59
Entacapone switchers vs. previously stopped at baseline	0.84	0.70	0.23	2.3	0.59	9.2

Among patients continuing opicapone beyond 6 months, there was a reduction in l‐dopa equivalent daily dosage (LEDD)^13^ from a mean at baseline of 899 mg (SD = 408) to a mean of 792 mg (SD = 296) at 6 months (*P* < 0.05). Among the group of patients who discontinued opicapone prior to 6 months, it was not possible to retrospectively quantify how many, and to what extent they had attempted to adjust their l‐dopa regime before discontinuing opicapone.

## Discussion

The aim of this audit was to identify whether opicapone may play a useful role in the management of PD patients with end‐of dose motor fluctuations who had not had adequate symptom control despite previous prescription of entacapone. These “real world audit data,” differ from the population that has been studied in randomized trials given their previous exposure to COMT inhibition, as well as because of differing inclusion–exclusion criteria. We found that a majority of patients responded well to opicapone and have a clinically relevant improvement of OFF time. However, our data also shows a high rate of opicapone intolerance leading to early treatment discontinuation.

This audit identified a higher proportion of patients who discontinued opicapone because of adverse events compared to the BIPARK 1 and 2 studies that reported adverse events associated discontinuation rates of 4.1% and 12%, respectively.^10^, ^12^ Patients in this audit had a similar age, gender distribution, disease duration, and baseline OFF time to those in BIPARK studies, however, differed in prior COMT inhibitor therapy (BIPARK studies excluded patients pre‐treated with entacapone whereas this audit only included patients in whom entacapone was not sufficiently beneficial or not tolerated). We found patients that had prior failure with entacapone before being considered for opicapone tended to be more likely to discontinue opicapone treatment than entacapone switchers.

The emergence of opicapone induced adverse events tended to occur within the first 3 months, with only a few individuals stopping opicapone after the 3‐month time point. The tolerability data and subsequent drug cessation demonstrates that continuation of opicapone is a highly self‐selecting process in that only 36 of 57 patients (63.2%) who started the drug, continued on it at 6 months. Among those who continued to use opicapone, there appears to be a clinically relevant impact on disability by reducing OFF time by ~2 hr per day, as assessed by interview. However, our data also found opicapone can aggravate hallucinations and psychosis and therefore, this drug should be used with caution in patients with previous or ongoing visual hallucinations or psychotic ideation. We found that patients who were taking entacapone up until the switch to opicapone were more likely to continue treatment beyond 6 months than those who had discontinued entacapone previously, possibly indicating that some individuals are less tolerant of any COMT inhibitor. Whether tolerability of the COMT inhibitors may be related to the Val158Met COMT polymorphisms as previously suggested^14^ requires further study.

There are a number of limitations to acknowledge in this audit:

1. The data are from a single tertiary center with a small sample size.
2. All clinicians prescribing opicapone in this study were aware of the need to tailor l‐dopa doses following initiation of opicapone. Although there was a mean reduction in LEDD among patients continuing opicapone at 6 months, patients who experienced new onset hallucinations immediately stopped opicapone with or without additional clinical advice. The extent to which other patients attempted to adjust their l‐dopa regime following onset of adverse events is not sufficiently reliably documented. It remains unclear whether earlier or more attentive adjustment to l‐dopa doses would have further improved opicapone tolerability among the group discontinuing the drug.
3. In patients who stopped opicapone because of non‐dopaminergic symptoms, it is unknown whether these complications are secondary to opicapone use or the progressive deteriorating nature of the disease.
4. The data reported here rely on self‐reporting from PD patients and are not confirmed by prospectively collected diary data, nor any other tool to assess severity and duration of motor fluctuations. This remains the method that most PD clinicians use to decide on adjustment or introduction of PD medications.
5. The absence of any placebo arm makes it impossible to estimate the placebo adjusted improvement in OFF time for patients who tolerated opicapone.
6. Among patients who had previously stopped entacapone, some individuals will have stopped it because of lack of efficacy, whereas others will have stopped entacapone because of adverse events. Our data do not capture whether opicapone is better suited to either of these subgroups although intuitively those stopping entacapone because of lack of efficacy might be more likely to continue opicapone than those stopping entacapone because of adverse events. This likely explains why our entacapone switchers were more likely to remain on opicapone.

Acknowledging these limitations, we conclude that opicapone can be a useful drug in the management of motor fluctuations in PD but caution should be exercised especially among patients with a history of neuropsychiatric features or those that have previously discontinued entacapone. Whereas patients with more advanced disease may well benefit from this drug, they should be treated more cautiously because of their increased chance of experiencing adverse events potentially leading to discontinuation. Because adverse events appear relatively soon after opicapone administration, review of patients within the first weeks after initiation of treatment should be a part of the clinical routine.

## Author Roles

(1) Research project: A. Conception, B. Organization, C. Execution; (2) Statistical Analysis: A. Design, B. Execution, C. Review and Critique; (3) Manuscript Preparation: A. Writing of the first draft, B. Review and Critique.

P.V.: 1C, 2B, 3B

A.B.: 1A, 1B, 1C, 2C, 3B

S.S.: 1A, 3B

L.S.: 1A, 3B

P.J.: 3B

K.B.: 1A, 3B

S.F.: 3B

T.S.: 3B

T.W.: 3B

R.W.: 3B

S.G.: 3B

P.L.: 3B

P.K.: 1A, 1B, 1C, 2A, 2C, 3B

T.F.: 1A, 1B, 1C, 2A, 2C, 3A, 3B

**Disclosures**

**Ethical Compliance Statement:** Verbal consent was obtained from all patients participating in this study. Formal; ethical approval was not obtained. We confirm that we have read the Journal's position on issues involved in ethical publication and affirm that this work is consistent with those guidelines.

Funding Sources and Conflicts of Interest: The authors report no funding sources for this work.

**Financial Disclosures for the previous 12 months:** T.F. has served on Advisory Boards for Bial, Neurocrine, and Living Cell technologies and has received honoraria for speaking at meetings supported by Bial, Profile Pharma, and Boston Scientific. P.V. and P.K. have received honoraria for speaking at meetings supported by Bial.
